# Hydrogel and Injectable Platelet-Rich Fibrin: A Synergistic Approach to Osteogenesis

**DOI:** 10.1055/s-0045-1809528

**Published:** 2025-06-23

**Authors:** Kent Sidharta, Suryono Suryono, Mardha Ade Pritia, Kwartarini Murdiastuti

**Affiliations:** 1 Clinical Dentistry Magister Program, Faculty of Dentistry, Gadjah Mada University, Yogyakarta, Indonesia; 2 Department of Periodontology, Faculty of Dentistry, Gadjah Mada University, Yogyakarta, Indonesia

**Keywords:** hydrogel, injectable platelet-rich fibrin, ALP activity, calcium deposition, bone dimension

## Abstract

**Objective:**

Injectable platelet-rich fibrin (i-PRF) features a higher concentration of growth factor and lower viscosity compared with PRF, making it advantageous for periodontal regenerative therapy. However, its low mechanical property and high degradation rate cause its limited usage in bone augmentation. Hydrogel interacts with i-PRF, which is expected to replace bone graft considering its disadvantages. Through the synergistic effects of the materials, a sustained release of growth factor is achieved, promoting bone formation and maturation.

**Materials and Methods:**

Osteogenic markers, including alkaline phosphatase (ALP) activity and calcium deposition, were measured at intervals of 1, 7, 14, and 21 days using osteoblast-like cells. In vivo study using the extraction socket of Wistar rat applied with the same material was also done and measured at 21 and 42 days. The study included three groups: hydrogel i-PRF, bone graft (FDBA) i-PRF, and a control (blank hydrogel) group. Measurements utilized ALP staining and Alizarin red S assays for the in vitro study and bone dimension for the in vivo study.

**Results:**

Hydrogel i-PRF significantly enhanced ALP activity on days 7 and 14 compared with the bone graft i-PRF and control groups ( *p* ≤ 0.05). Similarly, calcium deposition was notably higher in the hydrogel i-PRF group on days 14 and 21. Hydrogel i-PRF also preserves the bone dimension of the rat's extraction socket compared with bone graft i-PRF. These findings highlight the superior bone regeneration capacity of the hydrogel when combined with i-PRF, attributed to enhanced osteoblast proliferation, differentiation, and mineralization mediated by PDGF and BMP bound to collagen fibrils.

**Conclusion:**

Hydrogel with i-PRF exhibits improved osteogenic capability compared with bone grafts, showing promise as an alternative material for periodontal regenerative applications.

## Introduction

Periodontitis is a chronic microbial-origin infectious inflammation that leads to the destruction of periodontal tissues, which are the gingiva, cementum, periodontal ligament, and alveolar bone. Periodontal treatment is done for the formation of new attachments through the regeneration of lost tissue. 1 The regeneration is indicated by the improvement in clinical results, such as decreased pocket depth and bleeding on probing, and gain in clinical attachment level. 2 As compared with conventional modalities of treatment, regenerative procedures have been observed to result in more significant clinical, radiological, and histological advancements and deeper regeneration of cementum, periodontal ligaments, and bone.

Periodontal regeneration is achieved through tissue engineering, which has three main components: cell, biomaterial scaffolds, and bioactive signals, which encompass growth factors. 3 The material used to make the scaffold should be biocompatible with the ability to promote proliferation, differentiation, and tissue regeneration. Although autologous bone graft is considered the gold standard for scaffold material, it also has drawbacks that include limited supply and donor site morbidity, while allografts, such as freeze-dried bone allograft (FDBA), may cause immunological responses or even transfer pathogens. 4 Xenograft generates inferior bone quality and, thus, is linked with compromised primary stability following implant placement. 5

Collagen–chitosan hydrogels have been formulated as potential alternatives to bone grafts to surpass these drawbacks. The combination of collagen's osteogenic character and chitosan's stability by hydrogen bonding 6 in a porous three-dimensional scaffold facilitates cell migration and nutrient transport, hence improving bone formation. 7 Hydrogels also serve as drug delivery vehicles, providing the sustained release of bioactive molecules. Hydrogels, although useful, need to be reinforced with agents such as chitosan to increase their mechanical stability and reduce the rate of degradation.

Platelet concentrates, especially injectable platelet-rich fibrin (i-PRF), are extensively used in periodontal regeneration because of their accessibility, affordability, and lack of immunogenicity. Being a second-generation PRF, i-PRF is liquid in viscosity and contains higher levels of regenerative cells and growth factors, thus enhancing its regenerative capabilities. 8 This material has been utilized in a wide range of biomedical applications, including endodontic procedures, pulp capping, facilitation of orthodontic tooth movement, management of temporomandibular joint (TMJ) infections and osteoarthritis, facial aesthetics, hair regeneration, and as a vehicle for drug delivery, due to its advantageous properties such as biocompatibility, regenerative potential, ease of application, and sustained release capability. 9 Nevertheless, because i-PRF possesses low mechanical strength and degrades quickly, 10 it must be mixed with scaffolds like collagen–chitosan hydrogels to extend its osteogenic signaling.

Osteoblast-like cell was used to measure ALP activity and calcium deposition which convey osteogenic potential. ALP is an early marker of osteoblast formation, which promotes bone mineralization through the reduction of pyrophosphate, an inhibitor of hydroxyapatite formation. 11 12 Calcium deposition, observed during the later stages of differentiation, is routinely assessed by Alizarin red S staining, the gold standard method for the detection of calcium nodules in the extracellular matrix. The hydrogel's osteogenic potential can also be demonstrated in vivo by placing it in an extraction socket in *Rattus norvegicus* and assessing alterations in bone dimensions. The material is designed to reduce alveolar bone dimensional changes after tooth extraction, highlighting its regenerative capability with a focus on bone augmentation.

The purpose of this study was to investigate the influence of the i-PRF-mixed collagen–chitosan hydrogel on the proliferation of osteogenic cells and preservation of alveolar bone. This biomaterial enhances the activity of alkaline phosphatase (ALP) and induces calcium deposition in MG63 osteoblast-like cells, therefore potentially decreasing bone resorption following tooth extraction as compared with conventional bone grafting procedures.

## Materials and Methods

The in vivo and in vitro experiments were approved by the Gadjah Mada University Dentistry Faculty Research Ethical Commission (4/UN1/KEP/FKG-RSGM/EC/2024). The cells and animals were divided into three groups: the hydrogel i-PRF group, the bone graft i-PRF group, and a control group. FDBA was from the National Nuclear Energy Agency of Indonesia (BATAN).

### Collagen–Chitosan Hydrogel Composite

Chitosan was dissolved in 2% acetic acid for a period of one overnight. For complete dissolution, following this, the chitosan solvent was mixed with collagen powder and hydroxypropyl methylcellulose (HPMC) powder as a gelling agent and homogenized (Ultra-Turrax) overnight. A 25:75 ratio of collagen to chitosan, determined from an initial study, satisfied the pH and viscosity requirements needed for use. 13 The pH was checked using a pH meter and adjusted to a pH range of 6.2 to 7 with equal volumes of proper amounts of 1M NaOH.

### i-PRF Extraction and Processing

In the in vitro study, i-PRF was obtained from healthy male donors aged 18 to 30 years with normal platelet count. Informed consent was obtained first, and 10-mL sterile red-top vacutainer glass tube and a butterfly needle were used to collect the donor blood, which was centrifuged at 700 rpm for 3 minutes, 8 and it separated into three distinct layers: yellow, buffy coat, and red layer. 14 Red i-PRF was then collected from the buffy coat layer using a 3-mL syringe and an 18G needle.

For in vivo study, blood was drawn from the donor rat via a cardiocentesis procedure by a 20G needle inserted into the left ventricle, and a minimum of 10 mL of blood was drawn slowly and then centrifuged for 3 minutes at 700 rpm. Red i-PRF was drawn from the buffy coat layer using a 3-mL syringe and an 18G needle.

### Preparation of Hydrogel and Bone Graft

The i-PRF was blended with hydrogel and FDBA in a 1:1 ratio, to which the growth medium was added. The hydrogel for the control group was prepared with HPMC powder and distilled water only.

### Osteoblast-Like Cell (MG63)

Osteoblast-like cell line (MG63), purchased from ATCC, was plated in a 96-well microplate at a density of 2.5 × 10 ^3^ cells/well in DMEM with added antibiotic solution Pen Strep (Gibco) and 10% FBS. The cells were incubated at 37°C in 95% humidity and 5% CO _2_ overnight. The sample size was determined by the Federer formula, in which *n* is the sample size per intervention and *t* is the number of interventions; thus, ( *n* − 1) ( *t* − 1) > 15. Nine samples of each group were distributed to the cell culture and divided based on incubation times of 1, 7, 14, and 21 days.

### Measurement of ALP Activity

ALP activity assays were conducted with the ALP staining kit (SensoLyte) according to the instructions for use. On days 1, 7, 14, and 21, wash each well twice with the resulting solution. Add the appropriate volume of Triton solution to the wells and mix the solution again until homogenous. The solution was then placed in sterile microtubes and incubated with shaking at 4°C for a duration of 10 minutes. The mixture was then centrifuged for 10 minutes at 4,000 rpm under refrigerated conditions (velocity). Approximately 50 µL of the supernatant was then pipetted into each well, added to 50 µL of substrate, shaken for 30 seconds, incubated in the dark for 30 to 60 minutes, and read at 405 nm using a spectrophotometer.

### Calcium Deposition

The staining solution of Alizarin red S was prepared by mixing 2 mg of powder Alizarin red S (Merck) with 100 mL aquadest and shaking the solution for 15 minutes. Media was discarded after the incubation was done, and fixation of the cells was accomplished with formaldehyde at 4°C for 10 minutes. The cells were washed with PBS after fixation before staining with the prepared solution for 15 minutes. The wells were washed five times using deionized water afterward. For destaining, a 10% acetic acid and 20% methanol solution were added into each well and incubated for 15 to 20 minutes. Finally, the concentration of calcium was read at 405 nm using a spectrophotometer.

### Animal Preparation and Extraction Procedure

Twenty-four male Wistar rats (Rattus norvegicus) with body weights ranging from 250 to 300 g each were acclimatized in cages for 1 week before the experiment. The temperature and humidity were maintained at a comfortable 25°C and 50 to 70%, respectively. The teeth that were to be extracted were the left mandibular first molar. The anesthetic administered was Xylazine at 5 mg/kg. The rat's mouth was opened and held with an orthodontic power chain. A syndesmotomy was performed to separate the tooth from the supporting periodontal tissues. Gentle luxation using mosquito forceps continued until the tooth was completely extracted. The extraction sockets were dressed with different materials according to the experimental groups. Closure of the socket was done by suturing using Monofit 5/0 with an interrupted suturing technique. Ibuprofen at 40 mg/kg was given orally for 3 days as postoperative medication. The rats were euthanized using a lethal dose of Xylazine 40 mg/kg after either 21 or 42 days, depending on the group they were assigned to. Mandibles were removed for histopathological evaluation.

### Data Acquisition and Quantification

**Fig. 1:**
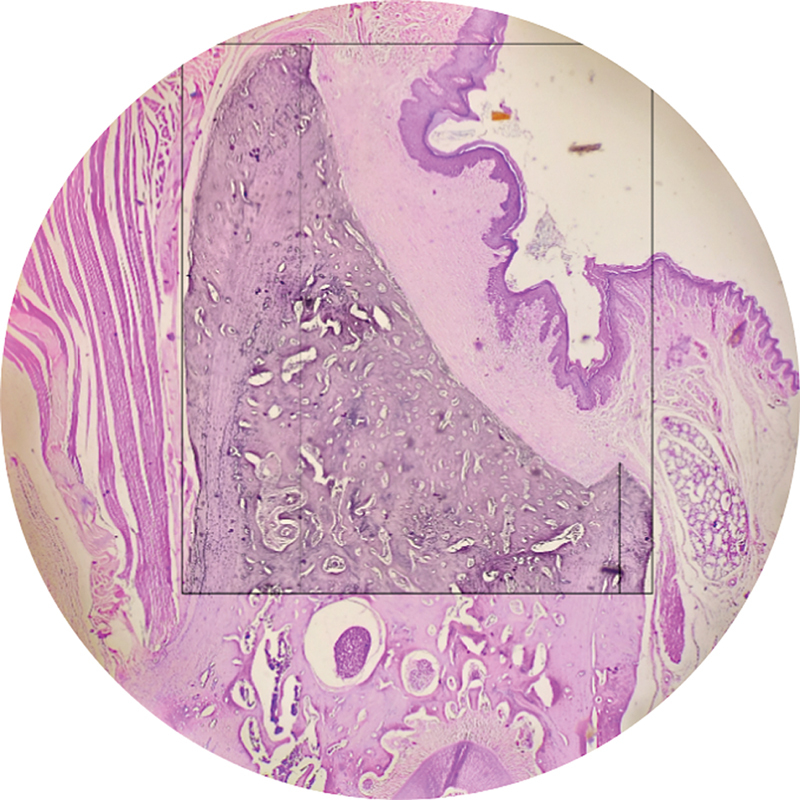
Reference lines of rat's socket histologic preparation.

The anatomical landmarks of the mandibles were located according to the previous study, 15 and two lines were drawn as references: T-Line, the most prominent point of the buccal bone; A-Line, a line perpendicular to the T-Line, intersecting the alveolar canal. The tips of the buccal and lingual bones were located, and two other perpendicular lines, named Bh-Line and Lh-Line, respectively, were drawn. All of these line measurements were accomplished with the Fiji ImageJ software. The volume of the alveolar bone was determined by the area of the bone demarcated by the A-Line and T-Line by utilizing the polygon tool in Fiji ImageJ. Additional measurements were taken for subsequent analysis ( Fig. 1 ).

### Statistical Analysis

Statistical analysis was conducted on SPSS version 29. All evaluations were tested for normality and homogeneity prior to running a two-way ANOVA. To determine significant group differences, a post hoc Tukey HSD test was used. A *p* -value < 0.05 was deemed statistically significant.

## Result

### Alkaline Phosphatase Activity

**Fig. 2:**
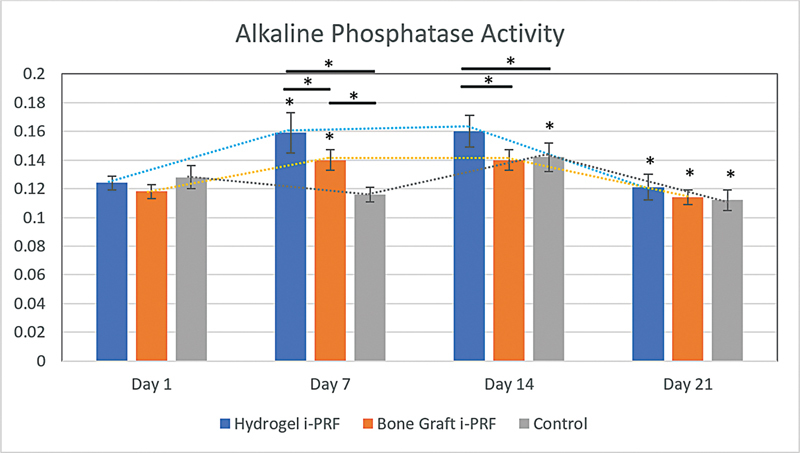
ALP activity based on treatment and incubation times.

On day 1, ALP activity revealed no significant difference among the groups, as indicated in Fig. 2 ( *p* = 0.994, *p* = 0.301). However, this value was significantly higher on day 7 in the hydrogel i-PRF group ( *p* ≤ 0.05) and the bone graft i-PRF group ( *p* ≤ 0.05). Surprisingly, both hydrogel i-PRF and bone graft i-PRF revealed significant difference compared with the control group ( *p* ≤ 0.05). On days 7 and 14, the ALP level of osteoblast in the hydrogel i-PRF group was significantly higher than that in the bone graft i-PRF group ( *p* ≤ 0.05). It was significantly higher than that in the control group ( *p* ≤ 0.05) as well. The activity of ALP did not differ between day 7 and day 14 for the hydrogel i-PRF ( *p* = 1.000) and bone graft i-PRF groups ( *p* = 1.000), whereas the control group showed a significant increase ( *p* ≤ 0.05). On day 21, the ALP levels in all groups significantly dropped ( *p* ≤ 0.05). The hydrogel group showed the highest ALP activity, but the difference was not statistically significant ( *p* = 0.843, *p* = 0.427). The ALP activity of osteoblasts in each group and their standard deviation are presented in Table 1 .

**Table 1:** Average ALP activity in osteoblasts with standard deviation

Incubation times	Hydrogel i-PRF	Bone graft i-PRF	Control
Day 1	0.124 ± 0.005	0.118 ± 0.005	0.128 ± 0.008
Day 7	0.159 ± 0.014	0.140 ± 0.007	0.116 ± 0.005
Day 14	0.160 ± 0.011	0.140 ± 0.007	0.142 ± 0.010
Day 21	0.121 ± 0.009	0.114 ± 0.005	0.112 ± 0.007

### Calcium Deposition

**Fig. 3:**
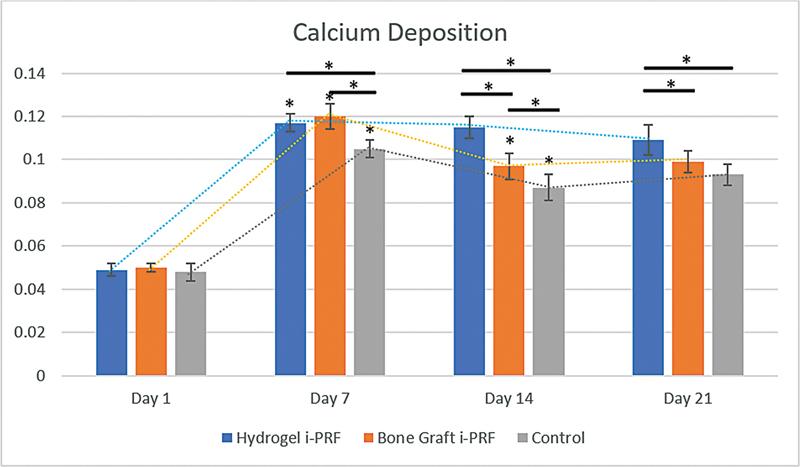
Calcium deposition based on treatment and incubation times.

**Fig. 4:**
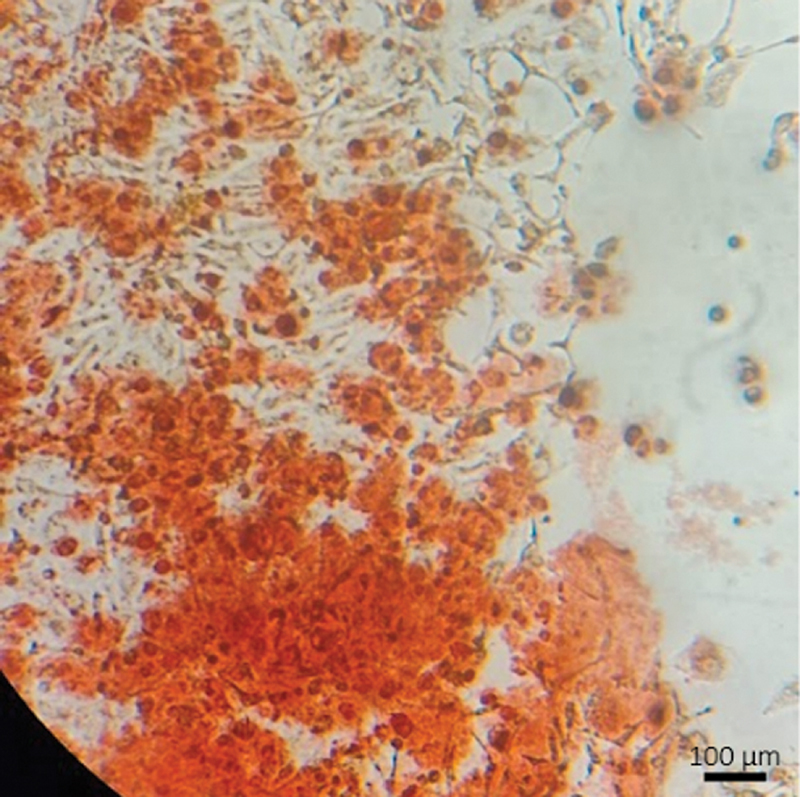
Calcium nodules in the hydrogel i-PRF group's well.

No significant differences were found among all groups at day 1, as indicated from Fig. 3 ( *p* = 1.000), while the mean and standard deviation of calcium deposition are presented in Table 2 . At day 7, a significant increase was observed for all groups, with the i-PRF-treated groups showing a significant increase when compared with the control group ( *p* ≤ 0.05). Mineral deposition within the hydrogel showed a considerable increase compared with the control group at days 7, 14, and 21 ( *p* ≤ 0.05) and compared with the bone graft group at days 14 and 21 ( *p* ≤ 0.05). The calcium deposition within the bone graft i-PRF group decreased significantly by day 14 ( *p* ≤ 0.05). Moreover, calcium nodules were observed in one of the wells of the hydrogel group on day 21, as indicated in Fig. 4 .

**Table 2:** Average calcium deposition in osteoblasts with standard deviation

Incubation times	Hydrogel i-PRF	Bone graft i-PRF	Control
**Day 1**	0.049 ± 0.003	0.050 ± 0.002	0.048 ± 0.004
**Day 7**	0.117 ± 0.004	0.120 ± 0.006	0.105 ± 0.004
**Day 14**	0.115 ± 0.005	0.097 ± 0.006	0.087 ± 0.006
**Day 21**	0.109 ± 0.007	0.099 ± 0.005	0.093 ± 0.005

### Buccal Bone Height

**Fig. 5:**
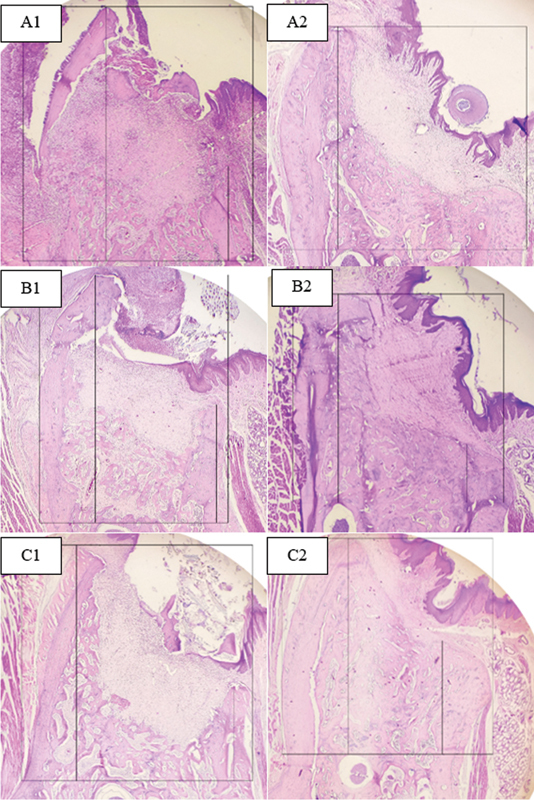
Bone height in the hydrogel group on (A1) day 21 and (A2) day 42, bone graft group on (B1) day 21 and (B2) day 42, and control group on (C1) day 21 and (C2) day 42.

**Fig. 6:**
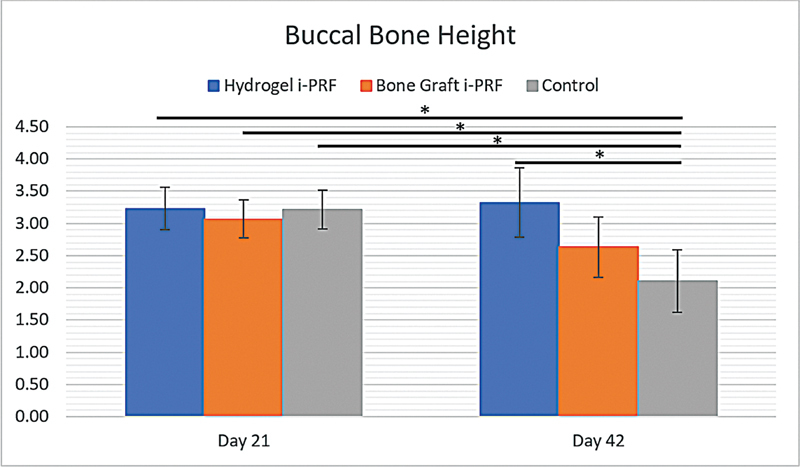
Mean and standard deviation of buccal bone height.

Buccal bone height was recorded on days 21 and 42 in the three experimental groups and shown in Table 3 . On day 21, there were no differences between the groups ( *p* > 0.05), with values of 3.230 ± 0.327 mm (hydrogel i-PRF), 3.070 ± 0.297 mm (bone graft i-PRF), and 3.213 ± 0.303 mm (control). By day 42, the hydrogel i-PRF and bone graft i-PRF groups exhibited significant increases ( *p* < 0.05) to 3.325 ± 0.539 mm and 2.633 ± 0.468 mm, respectively, when compared with the control groups with 2.103 ± 0.485 mm. This indicates greater preservation of buccal bone height ( Figs. 5 and 6 ).

**Table 3:** Mean and standard deviation of buccal bone height

Time point	*n*	Hydrogel i-PRF	Bone graft i-PRF	Control
**Day 21**	12	3.230 ± 0.327	3.070 ± 0.297	3.213 ± 0.303
**Day 42**	12	3.325 ± 0.539	2.633 ± 0.468	2.103 ± 0.485

### Lingual Bone Height

**Fig. 7:**
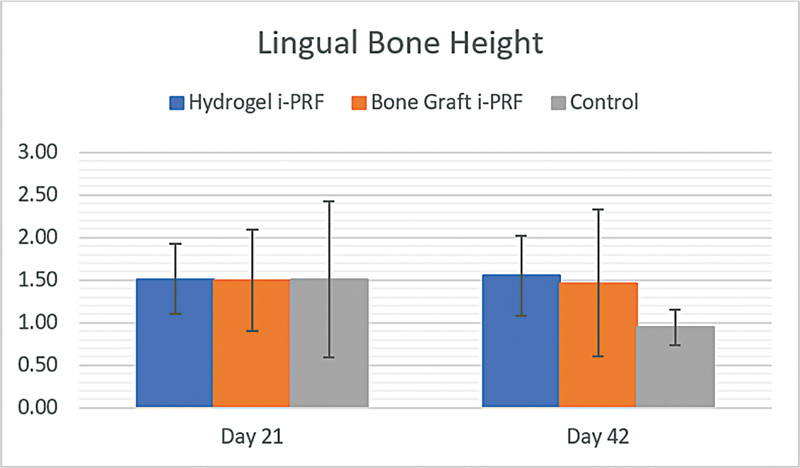
Mean and standard deviation of lingual bone height.

Regarding lingual bone height, the measurements were also taken at days 21 and 42 in the various groups presented in Table 4 . The height of the lingual bone on day 21 was comparable among groups, and no significant change ( *p* > 0.05) was noted: 1.513 ± 0.411 mm (hydrogel i-PRF), 1.498 ± 0.594 mm (bone graft i-PRF), and 1.510 ± 0.920 mm (control). At day 42, the hydrogel i-PRF group had the highest bone height ( *p* > 0.05) at 1.553 ± 0.469 mm, followed by the bone graft i-PRF and control groups at 1.468 ± 0.858 mm and 0.945 ± 0.211, respectively ( Fig. 7 ).

**Table 4:** Mean and standard deviation of lingual bone height

Time point	*n*	Hydrogel i-PRF	Bone Graft i-PRF	Control
**Day 21**	12	1.513 ± 0.411	1.498 ± 0.594	1.510 ± 0.920
**Day 42**	12	1.553 ± 0.469	1.468 ± 0.858	0.945 ± 0.211

### Total Alveolar Volume

**Fig. 8:**
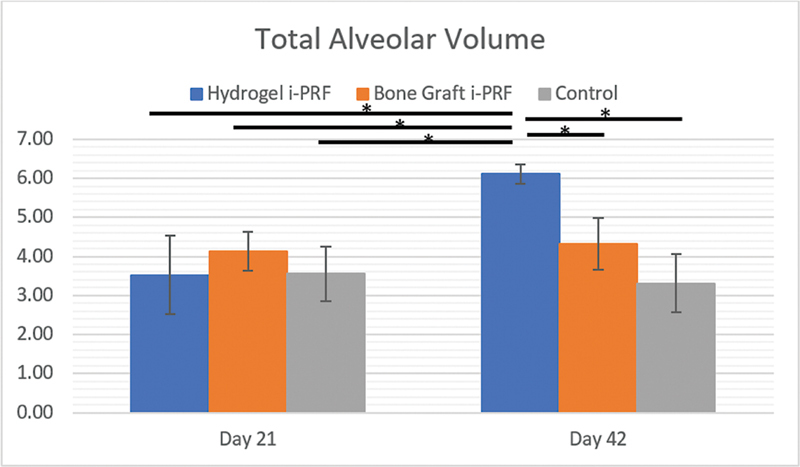
Mean and standard deviation of total alveolar volume.

The total alveolar volume was assessed at days 21 and 42 among the groups and shown in Table 5 . The alveolar bone volume presented significant differences among the groups throughout the periods. On day 21, all groups have the same alveolar bone volume ( *p* > 0.05) with the bone graft i-PRF group having the largest volume at 4.135 ± 0.496, followed by hydrogel i-PRF (3.528 ± 1.013) and control (3.553 ± 0.707). On day 42, the hydrogel i-PRF group showed the highest enhancement ( *p* < 0.05) at 6.113 ± 0.252, which was significantly greater ( *p* < 0.05) than that of the bone graft i-PRF (4.325 ± 0.659) and control (3.315 ± 0.741) groups ( Fig. 8 ).

**Table 5:** Mean and standard deviation of total alveolar volume

Time point	*n*	Hydrogel i-PRF	Bone graft i-PRF	Control
**Day 21**	12	3.528 ± 1.013	4.135 ± 0.496	3.553 ± 0.707
**Day 42**	12	6.113 ± 0.252	4.325 ± 0.659	3.315 ± 0.741

## Discussion

The hydrogel group showed superior osteogenic potential than the bone graft and control groups in both in vitro and in vivo studies. Higher ALP activity and calcium deposition in osteoblasts and greater alveolar bone height and volume in the rat's post-extraction socket were noted. The results indicate that hydrogel may be used as a potential alternative to allogenic bone graft for periodontal regeneration.

Although bone grafts, particularly FDBA, have been shown to be effective in regenerative procedures, 16 17 they have limitations, such as immune response risks, disease transmission, and donor site morbidity. Furthermore, bone grafts also show good clinical and radiographic quality but inferior histological outcomes because of the poor osteoinductive property. 18 In other research, it is shown that 48% of the patients who have been treated with allogenic bone grafts have sensitized immune systems that prolong the healing process. 19 On the contrary, hydrogel provides benefits like reduced immunogenicity, better interaction with i-PRF, and prolonged release of growth factors via collagen fibrils, which improve its regenerative capacity.

Collagen, which is known for its biocompatibility and bone-inducing properties, 20 activates osteoblast activity and favors extracellular matrix growth by enhancing the expression of ALP, as well as osteogenic mRNA and protein levels. 21 In combination with chitosan, it creates a porous hydrogel scaffold that can sustain cell migration and nutrient diffusion. This structural benefit guarantees that the contact between osteoblasts and bioactive molecules (i.e., i-PRF growth factors) is effective, leading to improved regeneration.

No statistically significant differences in the osteoblast ALP levels among the groups were detected on day 1, once again confirming that ALP acts as an early biomarker of osteoblastic activity, which is expressed only after 7 days in association with cellular proliferation. By day 7, ALP levels within the hydrogel and bone graft groups showed a significant elevation relative to the control group, representing the function of i-PRF in elevating osteoblast proliferation. Importantly, ALP activity within the hydrogel i-PRF group was significantly higher than that of the bone graft group, signifying the better scaffold characteristics of the hydrogel.

The stability of ALP activity on day 14 in both hydrogel i-PRF and bone graft i-PRF groups, in comparison to day 7, indicates that i-PRF is effective in sustaining ALP expression for a longer duration. It is speculated that ALP activity was the highest during days 7 and 14, which coincided with maximal collagen matrix deposition, a key event for the transition from the proliferative to the mineralization phase. 22 Yet, the hydrogel i-PRF group had greater ALP activity in comparison to all groups. This sustained activity is most likely caused by the collagen fibrils in the hydrogel, which regulate the growth factor release kinetics by restricting the diffusion of the growth factors via micro-pores. While growth factors directly released to collagen scaffolds typically exhibit a burst-release profile, PDGF-BB exhibits controlled release that tracks the gradual degradation of the collagen matrix. 23

By day 21, ALP activity had decreased considerably in all groups, with no measurable differences between them, and the levels basically returned to baseline levels seen on day 1. During this terminal differentiation stage, osteoblasts become confluent and mature, and some of them undergo apoptosis. The formed calcium nodules that are deposited in the wells are markers of mineralization. For extracellular matrix mineralization, calcium ions are sequestered in the form of granules in mitochondria or matrix vesicles and mobilized as essential sources of calcium and phosphate necessary for hydroxyapatite formation. 24

Calcium deposition on day 1 did not differ significantly between groups, a reflection that it is a marker of late osteoblast differentiation that had not yet occurred. By the seventh day, calcium deposition was significantly higher than it had been on day 1. Both the hydrogel and bone graft groups showed considerably greater calcium deposition than the control group, echoing the ALP activity observed on day 7. The enhanced calcium nodule formation observed with i-PRF further supports its ability to promote calcium deposition by upregulating osteoblast differentiation transcription factors and osteogenic genes such as RUNX2, while concurrently downregulating osteogenic inhibitors. 25

At day 14, the hydrogel i-PRF group's calcium levels remained comparable to day 7, showing the highest concentration of calcium compared with all groups. This finding points to the greater mineralization potential of the hydrogel scaffold compared with allogenic bone grafts. Conversely, the i-PRF group treated with allogenic bone graft exhibited a noticeable reduction in calcium deposition on day 14, indicating its lack in maintaining mineralization at the later stages of osteoblast differentiation. The levels of calcium deposition had already reached stabilization on day 21, while the hydrogel group still presented the highest content of calcium with significant differences from the other groups. The results obtained indicate a prolonged release of growth factors from the collagen–chitosan hydrogel, confirming previous studies 26 27 and promoting mineralization during the later stages of osteoblast differentiation

The analysis of the height and volume of the rat's post-extraction sockets at 21 and 42 days is key to determining the efficacy of hydrogel with i-PRF as a regenerative material. No differences in the buccal or lingual bone height among the groups were found at day 21, which indicates that the initial phase of bone healing could be similar regardless of treatment. The shape of the extracted tooth was still recognizable at this time point, pointing to limited buccal and lingual bone resorption. On day 42, the hydrogel exhibited higher buccal and lingual bone heights than the allogenic bone graft and control group. Notably, the bone heights in this group were more or less the same from days 21 to 42, which underscores the capacity of the material to maintain bone dimensions over the long term. Lingual bone height did not show any significant differences among all groups. This finding is consistent with the previous study 28 that reported greater bone resorption on the buccal aspect compared with the lingual aspect, likely due to differences in bone thickness. The thinner buccal bone is more susceptible to resorption, resulting in a residual ridge that slopes from the lingual to the buccal side.

Conversely, in the control group, where no interventions were made for bone height maintenance, there was a high resorption by day 42. The buccal bone height decreased by ∼1.11 mm (34.5%), and lingual bone height decreased by 0.56 mm (37.4%). These results highlight the imperative role played by bone preservation materials in reducing post-extraction bone loss. The gradual infilling of the extraction socket with bone in the hydrogel i-PRF group also demonstrates the regenerative capability of this scaffold through collagen, which facilitates sustained growth factor release and better cell attachment, enhancing osteoblast proliferation, differentiation, and overall osteogenesis. 26

In terms of alveolar bone volume, all groups showed similar findings on day 21 ( *p* > 0.05), which may be attributed to the minimal early resorption at this phase. On day 42, the hydrogel i-PRF group showed a significant and consistent increase in bone volume, which was greater than that of the bone graft i-PRF group. This notable improvement may be due to the gradual filling up of the socket space with new bone and bone marrow formation, leading to augmented volume. The hydrogel's porous architecture is essential in tissue engineering scaffolds, as it ensures sufficient surface area for cellular adhesion and proliferation, while also enabling optimal diffusion of nutrients and metabolic waste. 27 Conversely, the control group consistently exhibited the lowest bone height and volume readings, thereby highlighting the natural constraints imposed by untreated healing. The bone graft i-PRF group showed a gradual decline in bone height and volume by day 42, which indicates its lesser capacity to maintain osteogenic signaling over an extended period. These findings point to the hydrogel's greater regenerative capacity, which not only inhibits bone resorption but actually increases bone regeneration, making it a very appealing option compared with conventional grafting materials.

Hydrogel is inherently antimicrobial, which is an added advantage compared with conventional bone grafts. 29 The blend of collagen and chitosan produces a biomaterial with excellent antimicrobial activity against a wide range of pathogenic microorganisms through the cationic nature of chitosan, which binds to microbial cell membranes, destabilizing and killing the cells. 30 The antimicrobial properties minimize the risk of postoperative infection, which is a frequent complication associated with bone regeneration surgery. Conventional bone grafts, especially allografts and xenografts, do not possess such antimicrobial properties and can necessitate further procedures, including antibiotic coatings, to reduce infection threats. The antimicrobial quality of collagen–chitosan provides a cleaner environment for healing, enhancing tissue regeneration at a more effective rate. Another essential benefit of hydrogel is the controlled degradation schedule. Collagen–chitosan hydrogel retains its structure while facilitating tissue regeneration, with full degradation taking place around 6 weeks after surgery. 31 This duration is consistent with the critical stages of bone healing, allowing the scaffold to provide mechanical support and act as a reservoir for growth factors in the initial stages of osteogenesis, while being slowly resorbed as the new bone is deposited. Conversely, conventional allograft and xenograft tend to show inconsistency in degradation rates, leading to extended persistence or premature resorption, which can interfere with bone healing and remodeling. 32

The promising osteogenic activity exhibited by collagen–chitosan hydrogel indicates its application as an alternative to allogenic bone grafts, owing to the latter being implicated in immune sensitization along with other disadvantages. This biomaterial could find particularly useful application in dental procedures like ridge preservation, where it is important to preserve alveolar bone size after tooth extraction. However, additional studies are warranted to assess its impact on other animal models and regeneration markers, its mechanical strength and degradation profile, to justify its benefits over conventional bone grafting biomaterials.

Future research should focus on ensuring that the hydrogel system maintains sufficient flexibility to integrate with advanced forms of platelet concentrates. Concentrated PRF (C-PRF), characterized by its higher cellular content and elevated concentrations of growth factors, 8 represents a promising innovation despite the limited number of studies currently available. However, its use is associated with certain limitations, such as the requirement for a horizontal centrifuge system, which may not be readily available in all clinical or laboratory settings. Therefore, evaluating the compatibility of our hydrogel with this novel liquid PRF formulation is an important step for future development.

## Conclusion

This research posits that, within its constraints, the blend of hydrogel and i-PRF has greater osteogenic capability compared with allogenic bone grafts in MG-63 osteoblast-like cells for earlier and more efficient bone generation. Such materials also maintain the socket dimensions of the extraction in rats and are responsible for increasing the volume of the extraction socket. Further research involving different animal models and other markers of regeneration needs to be conducted to enable more definitive findings in human studies.
